# The American Antiquarian and Oriental Journal

**Published:** 1897-02

**Authors:** 


THE AMERICAN ANTIQUARIAN AND ORIENTAL JOURNAL. Address Stephen D. Peet, Editor, 175 Wabash Avenue, Chicago, Ill., or Good Hope, Ill.

THE AMERICAN ANTIQUARIAN AND ORIENTAL JOURNAL begun its XIXth Volume in January, 1897. The volume will contain, among other things, a series of articles on the Ancient Cities of Central America, by Dr. Maler, the distinguished archæologist of Germany; also a series of articles on the Cliff Dwellings of Arizona and New Mexico, both fully illustrated. Dr. Cyrus Thomas will continue his articles on Indian Migrations. Notes on recent discoveries in Egypt, by Dr. Wm. C. Winslow, D. D., of Boston; and on the Holy Land, by Prof. T. F. Wright, of Cambridge; on European Archæology, by Dr. D. G. Brinton.

It is the design of this magazine to furnish information on archæology in all lands, so that it will be especially useful to those who take only one archæological journal. $4.00 per year.

